# Supplementary material for: Noninvasive imaging-guided ultrasonic neurostimulation with arbitrary 2D patterns and its application for high-quality vision restoration
Source: Nat Commun. 2024 May 27;15:4481. doi: 10.1038/s41467-024-48683-6 (PMC11130148; doi:10.1038/s41467-024-48683-6)
Supplement: Supplementary file 1 — Supplementary information [file 41467_2024_48683_MOESM1_ESM.pdf]

Supplementary Information

Supplementary Note 1: Eye movement tracking and delay.

Tracking eye movement accurately is critical for precise retinal stimulation, given the significant variability in eye movement velocity across different modes[1]. For instance, eye velocity can remain at 0°/s for 50-600 ms in fixation mode[2], below 30°/s in smooth pursuit mode, and surge to as high as 700°/s in saccades mode. Furthermore, involuntary movements[3] like microsaccades, drift, and tremor, though minor (up to 1°), also play a role.

The main contributors to delay in the retina stimulation system are the algorithms for spatial correction detection and pattern generation with spatial correction feedback. Our algorithms demonstrate efficiency, with processing times under 50 ms—less than 35 ms for edge detection and under 15 ms for pattern generation—on our system (MATLAB, Intel i7-7700K @ 3.60 GHz). This results in an angular error of up to 1.5° in smooth pursuit mode and 35° in saccades mode. Given that involuntary eye movements are barely perceptible even in healthy individuals, an angular error of 1.5° should be deemed acceptable for patients using visual prostheses. We acknowledge that the angle error is more pronounced in saccade mode. However, patients with acquired blindness—who represent the primary demographic for retinal prostheses and have typically lost photoreceptor function in adulthood—are capable of controlling their eye movements similarly to sighted individuals. They can consciously minimize or completely avoid saccadic movements while using the ultrasonic retinal prostheses in their daily activities[4,5].

In conclusion, standard eye movements are unlikely to pose significant issues for the functionality of ultrasonic retinal prostheses. The acceptable range of angular error (1.5°) and the potential for further improvement with contact lens design ensure that eye movement does not compromise the effectiveness of these devices.

Reference:

1 Land, M. & Tatler, B. Looking and acting: Vision and eye movements in natural behaviour. (Oxford University Press, 2009).

2 Hessels, R. S., Niehorster, D. C., Nyström, M., Andersson, R. & Hooge, I. T. Is the eye-movement field confused about fixations and saccades? A survey among 124 researchers. *Royal Society open science* **5**, 180502 (2018).

3 Martinez-Conde, S., Otero-Millan, J. & Macknik, S. L. The impact of microsaccades on vision: towards a unified theory of saccadic function. *Nature Reviews Neuroscience* **14**, 83-96 (2013).

4 Schneider, R. M. *et al.* Neurological basis for eye movements of the blind. *PloS one* **8**, e56556 (2013).

5 Leigh, R. & Zee, D. S. Eye movements of the blind. *Investigative Ophthalmology & Visual Science* **19**, 328-331 (1980).

Supplementary Note 2: Acoustic attenuation in human eyeball.

Given the significant difference between the human eyeball (diameter around 24 mm) and the rat eyeball (diameter around 7 mm), a common concern is that the acoustic attenuation in the human eyeball will be significantly stronger than the attenuation in the rat eyeball. This attenuation and attenuation-related heating effect over the long distance will make ultrasound retina prostheses infeasible for human applications, especially for high-frequency ultrasound.

Here we performed FEM simulations at 20 MHz (1.1 MPa, 15 ms duration) to quantify the acoustic attenuation and ultrasound-induced heat generation in human eyeballs, demonstrating that acoustic attenuation in human eyeball is still relatively low and the heating effect won't be a concern. As shown in Fig. S1a&b, the acoustic pressure at the retina location is around 1.3 MPa in free field and 1.1 MPa with the attenuation in human eyeball, which indicates a 1.45 dB attenuation. Accordingly, Fig. R1c shows the temperature increase in the human eyeball under 20 MHz ultrasound stimulation is still within 1 degree.

The key reason leading to this counterintuitive phenomenon is the significantly low acoustic attenuation of the vitreous (70-100 times lower than other tissues), which fills the main volume of the human eyeball. As shown in our updated Table S2, acoustic attenuation of aqueous and vitreous is 0.01 – 0.012 dB/cm/MHz and 0.78 – 1.19 dB/cm/MHz in other tissues. Given the average structure of human eyeball, we can do a quick estimation of the attenuation distribution in the human eyeball with minor mathematical simplifications:

Attenuation from aqueous and vitreous = 0.012 dB/cm/MHz * 20 MHz * 2 cm = 0.48 dB

Attenuation from sclera and iris = 1.0 dB/cm/MHz * 20 MHz * 0.1 cm = 2 dB

Attenuation from lens = 1.2 dB/cm/MHz * 20 MHz * 0.4 cm = 9.6 dB

Since rat eyeball has a relatively big lens with a center thickness of around 6 mm, which is even thicker than human's lens (~ 4 mm), the overall acoustic attenuations in rats' eyeball and humans' eyeball are similar.

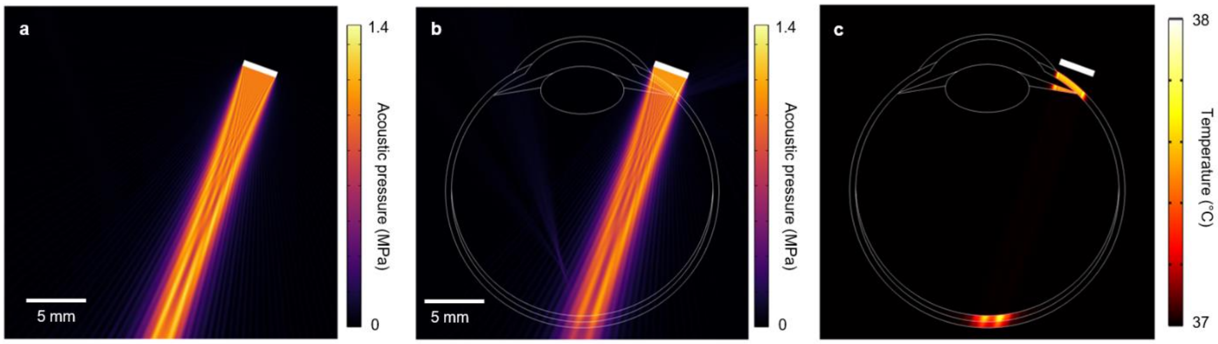

Supplementary Fig. 1. Acoustic attenuation and heat generation of 20-MHz ultrasound in the human eyeball. **a,** Ultrasound field in free space. **b,** Ultrasound field in the human eyeball with attenuation. **c,** Ultrasound-induced temperature increase distribution in the human eyeball.

Table S1. Materials and parameters of 4.5-MHz ultrasound array.

Layer	Material	Thickness
**Piezoelectric**	PZT-4	313 µm
**First Matching layer**	2-3 um Silver/Epoxy composite	103 µm
**Backing**	E-solder	5 mm
**Coating**	Parylene C	15 µm

Table S2. Parameters of tissues in the eye for acoustic and thermal simulation[1,2].

Tissues	**Density (kg/m^3)**	**Sound speed (m/s)**	**Heat capacity at constant pressure (J/kg/K)**	**Thermal conductivity (W/m/K)**	**Attenuation (dB/cm/MHz)**
Water	1000	1500	4178	0.62	0
Cornea	1062	1586	4178	0.58	0.78
Aqueous	1007	1497	3997	0.59	0.01
Vitreous	1005	1532	3999	0.6	0.012
Lens	1076	1647	3000	0.40	1.19
Iris*	1090	1588	3421	0.49	0.62
Sclera	1088	1647	4178	0.58	0.97
Retina	1034	1538	3680	0.57	1.15

*Direct measurement results of Iris are not found. Muscle parameters were used for iris since iris mainly composed of muscle[2].

Reference:

1. Nabili, M., Geist, C. and Zderic, V., 2015. Thermal safety of ultrasound-enhanced ocular drug delivery: A modeling study. Medical physics, 42(10), pp.5604-5615.
2. Asano, N., Schlötzer-Schrehardt, U. and Naumann, G.O., 1995. A histopathologic study of iris changes in pseudoexfoliation syndrome. Ophthalmology, 102(9), pp.1279-1290.

Supplementary Figures

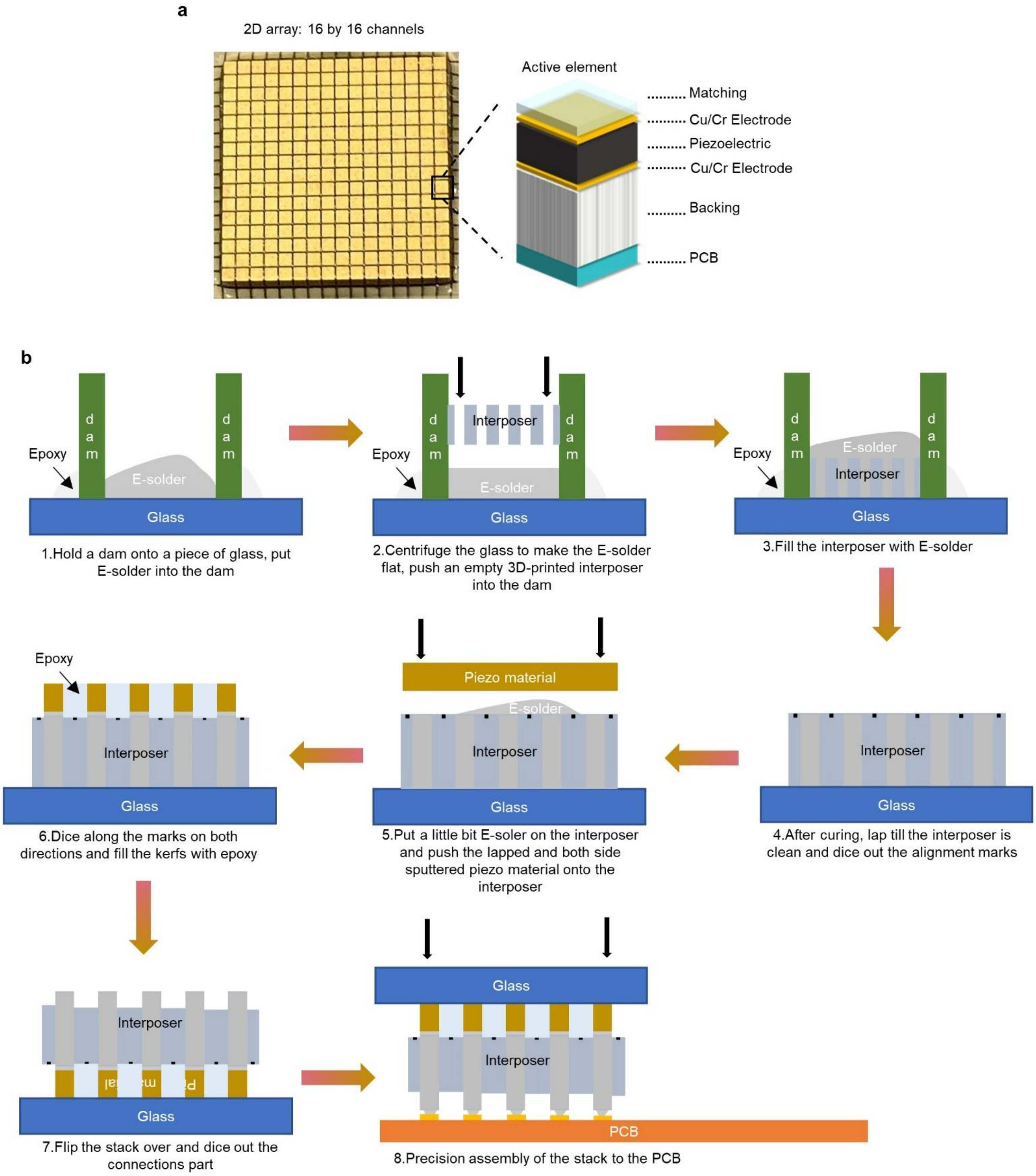

Supplementary Fig. 2. The fabrication of ultrasound 2D array. **a,** A photo of fabricated ultrasound 2D array and the detailed structures of each layer. **b,** The fabrication process of ultrasound 2D array.

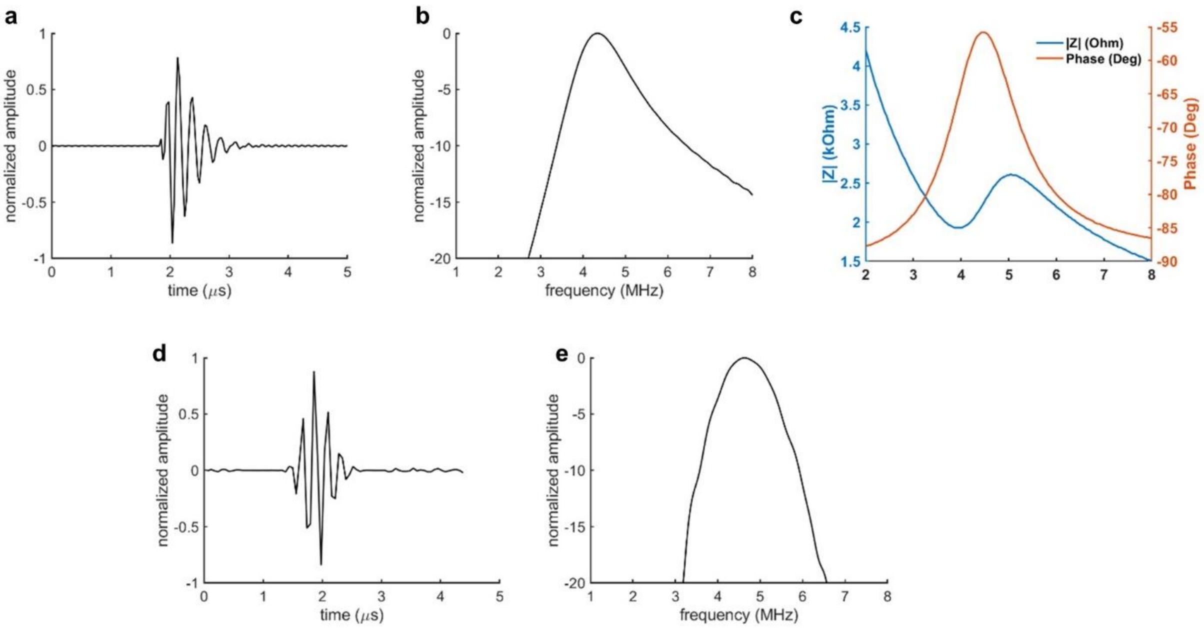

Supplementary Fig. 3. The characterization of a representative element in ultrasound 2D array. **a,** Simulated pulse-echo response of a representative element in the array. **b,** Simulated frequency spectrum of a representative element in the array. **c,** Measured electric impedance curve of a representative element in the array. **d,** Measured pulse-echo response of a representative element in the array. **e,** Measured frequency spectrum of a representative element in the array.

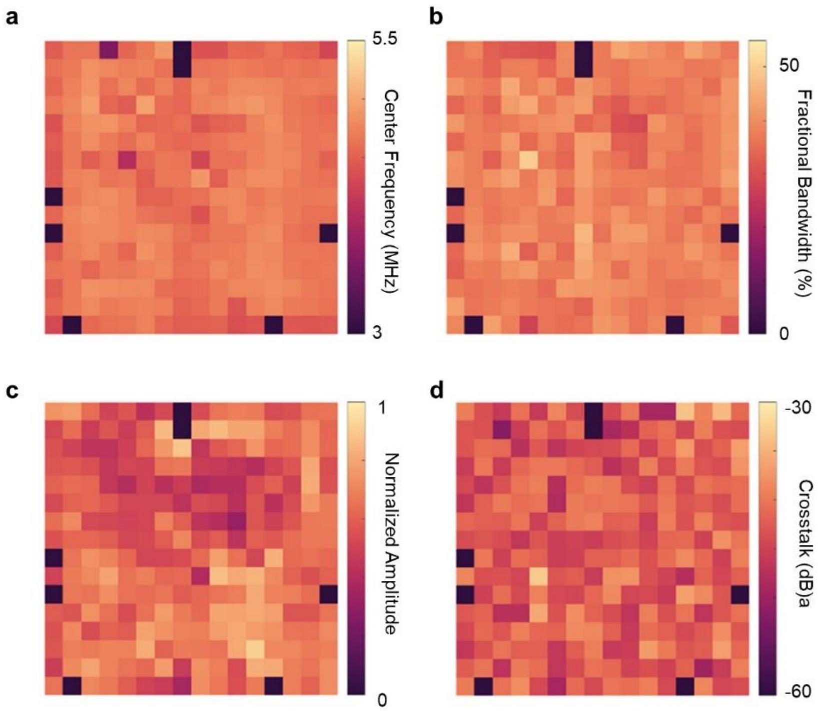

Supplementary Fig. 4. The characterization of all elements in ultrasound 2D array. **a,** Measured distribution of center frequency of all elements in the array. **b,** Measured distribution of fractional bandwidth of all elements in the array. **c,** Measured distribution of normalized pulse amplitude of all elements in the array. **d,** Measured distribution of crosstalk of all elements in the array.

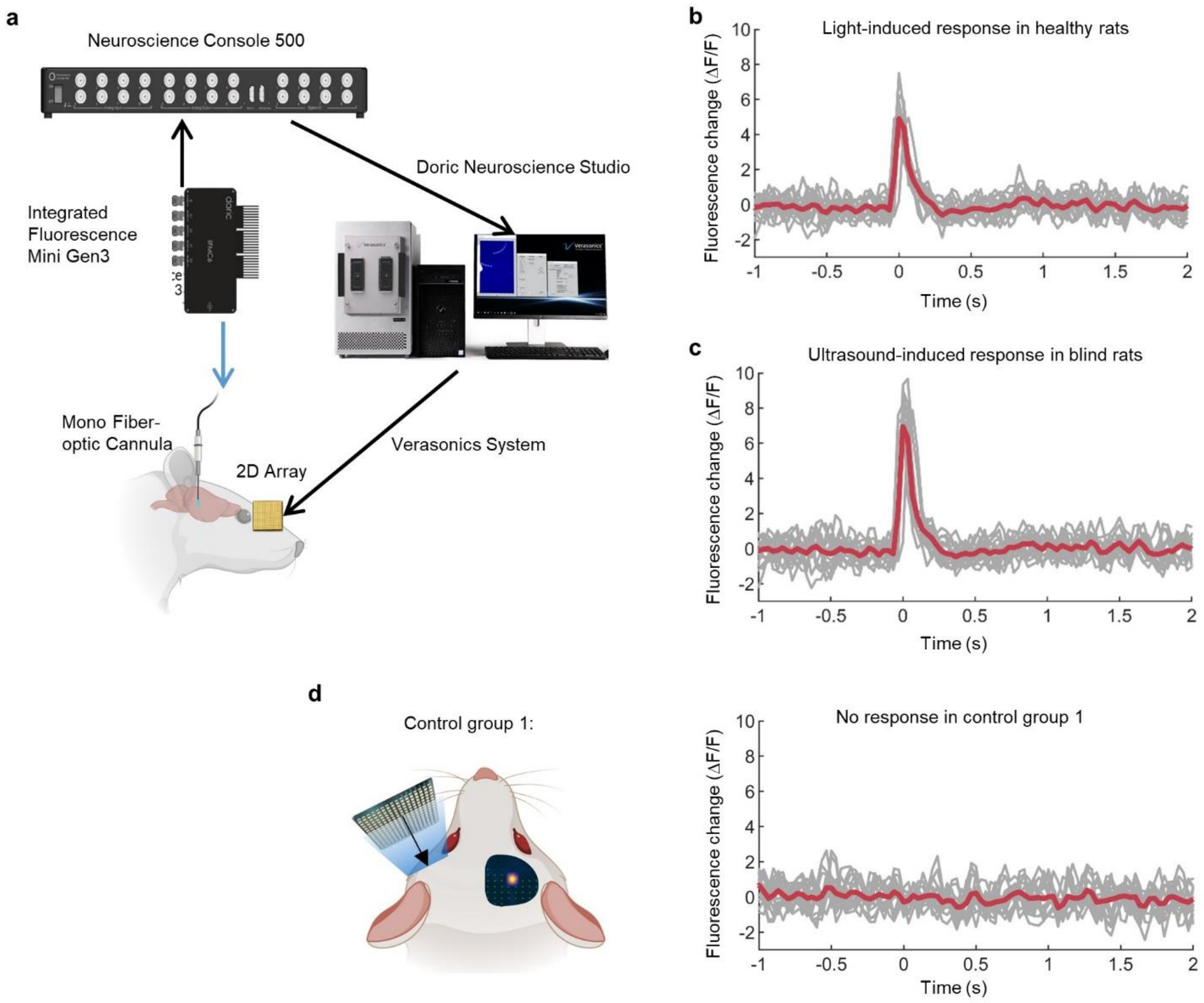

Supplementary Fig. 5. In vivo fiber photometry showed ultrasound-evoked visual signals in visual cortex. **a,** The diagram of in vivo fiber photometry experiment setup. **b,** Light-induced neuron response in healthy rats. **c,** Ultrasound-induced neuron response in PD rats. **d,** In the control group 1, ultrasound stimulation was tilted to focus on between the eye and ear. No neuron response in control group 1 (blind rats).

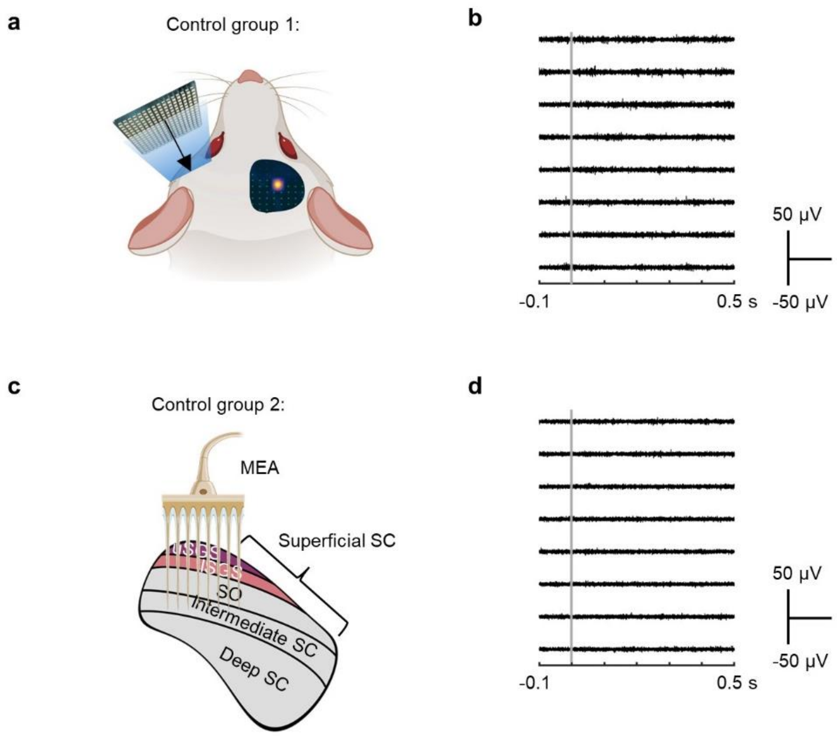

Supplementary Fig. 6. Control groups show no response. **a,** Schematic of Control group 1: ultrasound stimulation was tilted to focus on between the eye and ear of blind rats. **b,** No neuron response was observed from the control group 1. **c,** Schematic of Control group 2: MEA was inserted 1.5 mm deeper than the surface of SC. **d,** No neuron response was observed from the control group 2.

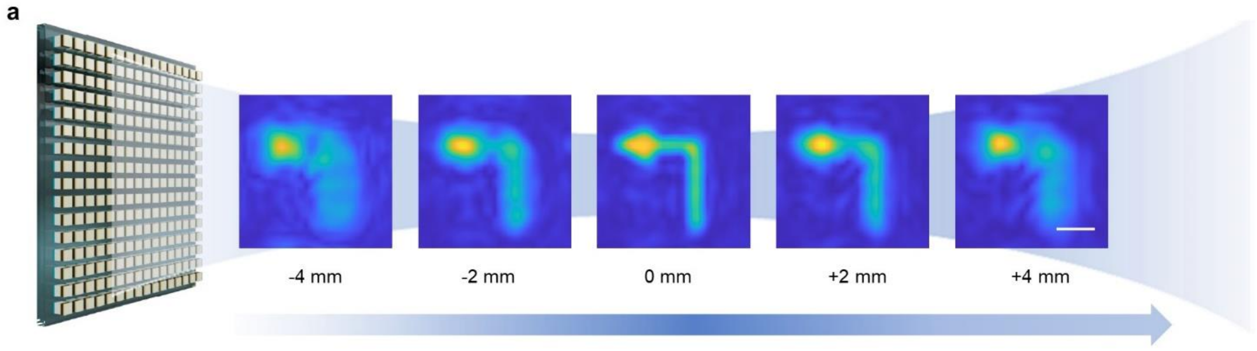

Supplementary Fig. 7. **a,** Hydrophone-measured ultrasound field distribution in different depths. “0 mm” indicates the depth of the targeted stimulation plane. White bars indicate the length of 1 mm.

a

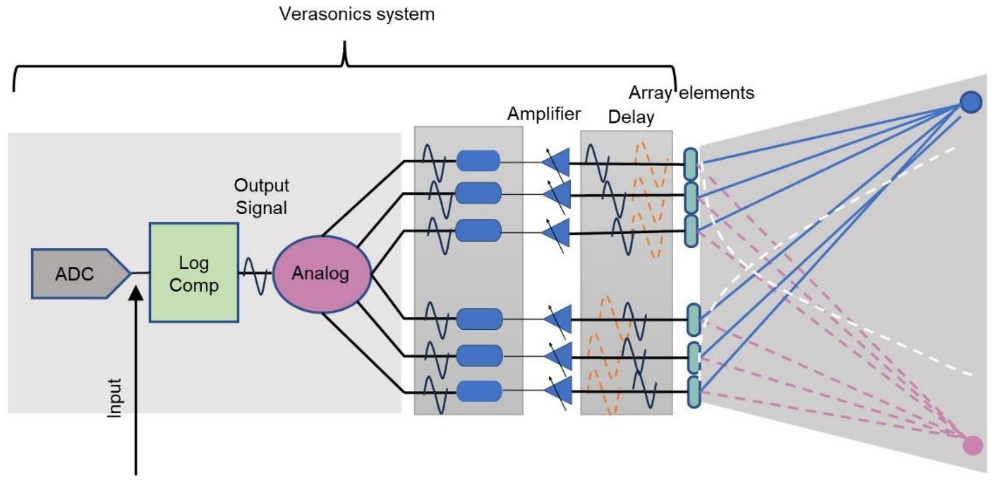

Supplementary Fig. 8. **a,** The schematic diagram of Verasonics to demonstrate the control of amplitude and delay of each element in the ultrasound array.

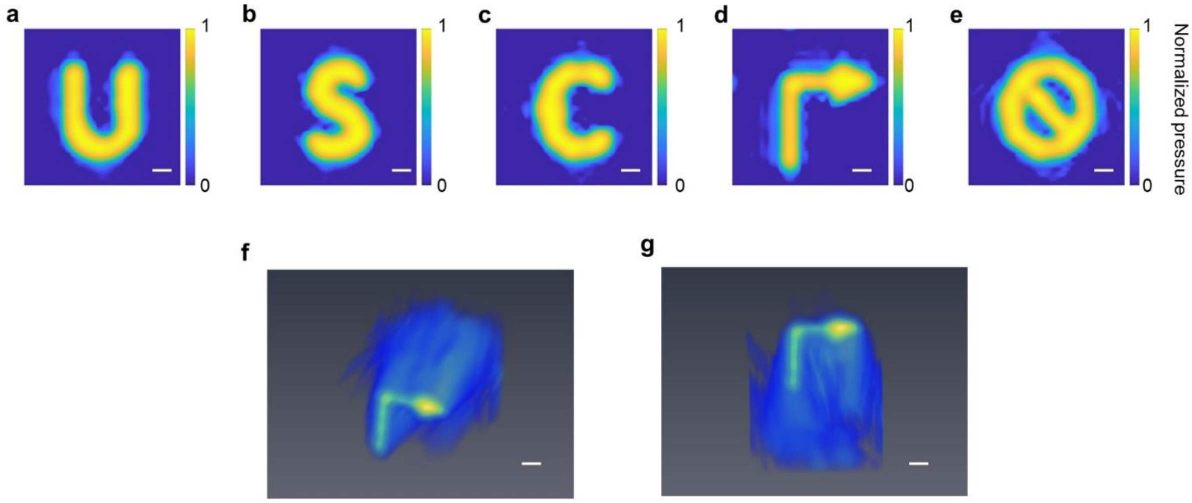

Supplementary Fig. 9. **a-e,** The simulated ultrasound field distribution generated by ultrasound 2D array of letters “U”, “S”, “C” and symbols for “Turn right”, “Stop”. **f&g,** 3D reconstruction of generated ultrasound field.

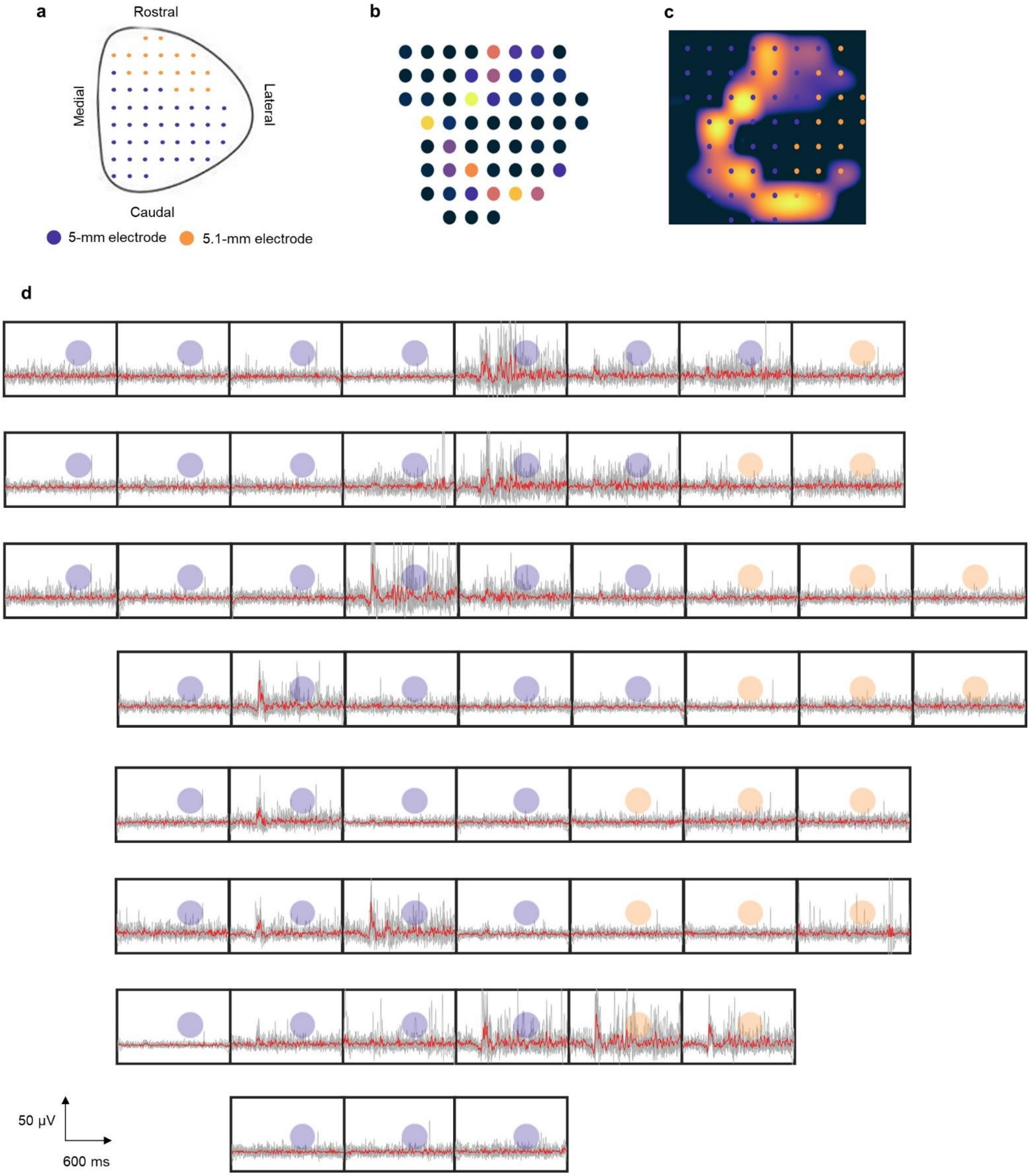

Supplementary Fig. 10. **The response mapping of a representative pattern, letter "C". a,** The spatial relation between the customized MEA and the surface of SC. **b,** The raw response mapping from each electrode of MEA. **c,** The interpolated response mapping. **d,** The signals from each electrode of the MEA. Gray lines indicate the raw signal is each recording and red lines indicate the averaged signal.

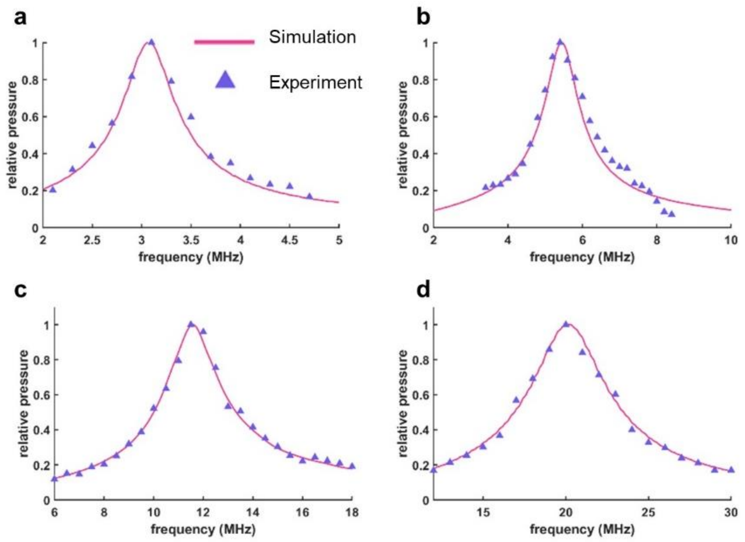

Supplementary Fig. 11. Frequency spectrum of ultrasound transducers at different frequency. **a,** The frequency spectrum 3-MHz ultrasound transducer. **b,** The frequency spectrum 5.4-MHz ultrasound transducer. **c,** The frequency spectrum 12-MHz ultrasound transducer. **d,** The frequency spectrum 20-MHz ultrasound transducer.

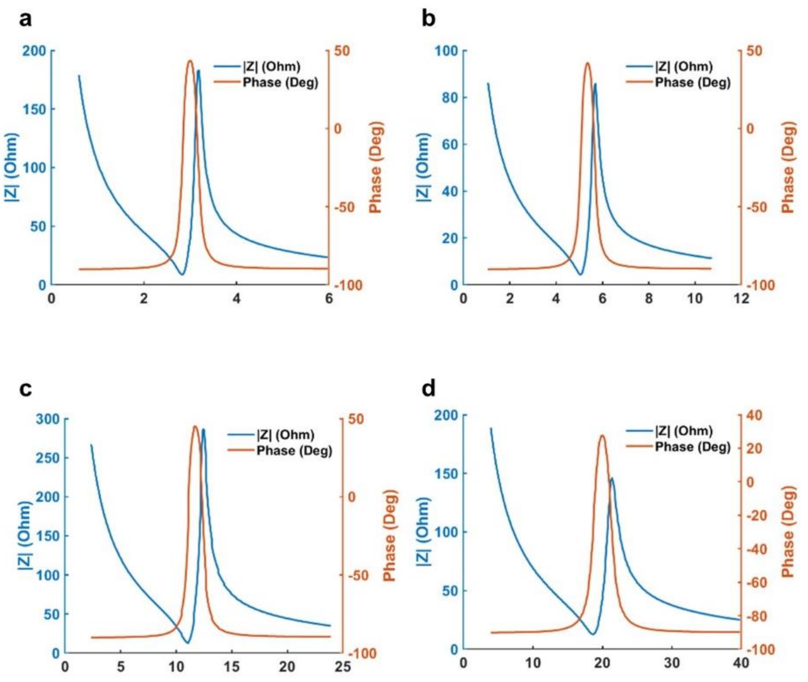

Supplementary Fig. 12. Electric impedance of ultrasound transducers at different frequency. **e-f,** Electric impedance of transducers with center frequency of 3, 5.4, 12, and 20 MHz.

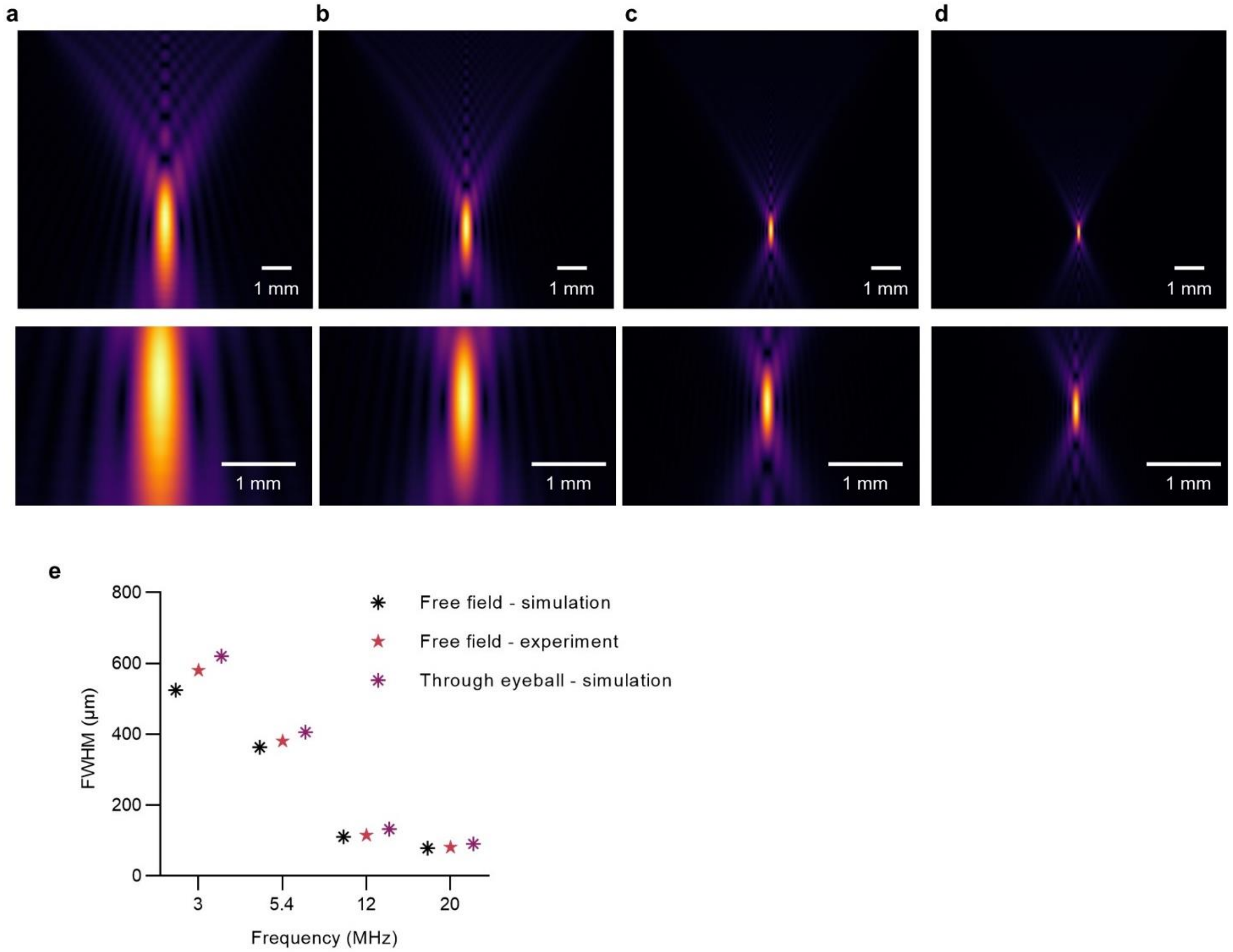

Supplementary Fig. 13. Frequency-dependent resolution of ultrasound. **a,** Top: ultrasound field distribution in x-z plane of 3-MHz ultrasound. Bot: the zoom-in view of focus. **b,** Top: ultrasound field distribution in x-z plane of 5.4-MHz ultrasound. Bot: the zoom-in view of focus. **c,** Top: ultrasound field distribution in x-z plane of 12-MHz ultrasound. Bot: the zoom-in view of focus. **d,** Top: ultrasound field distribution in x-z plane of 20-MHz ultrasound. Bot: the zoom-in view of focus. **e,** The summary of spatial resolution in different conditions: free-field simulation, free-field hydrophone measurement, and through-eyeball simulation. Source data are provided as a Source Data file.

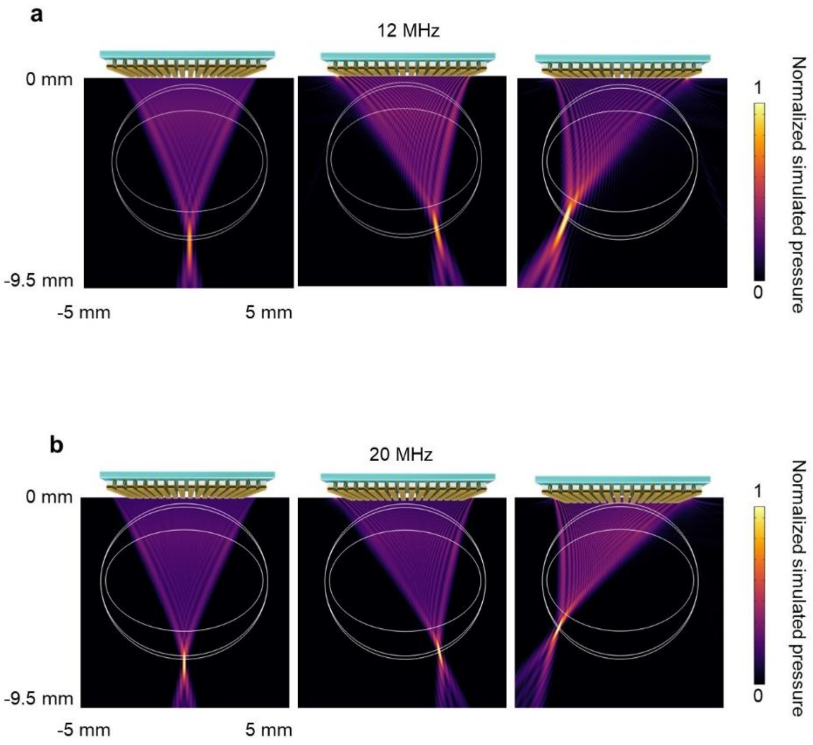

Supplementary Fig. 14. Steerable ultrasound focus can cover the whole retina. **a,** the simulated scanning 12-MHz ultrasound fields in x-z plane. **b,** the simulated scanning 20-MHz ultrasound fields in x-z plane. Left: 0 mm; Middle: 1.5 mm; Right: -3 mm.

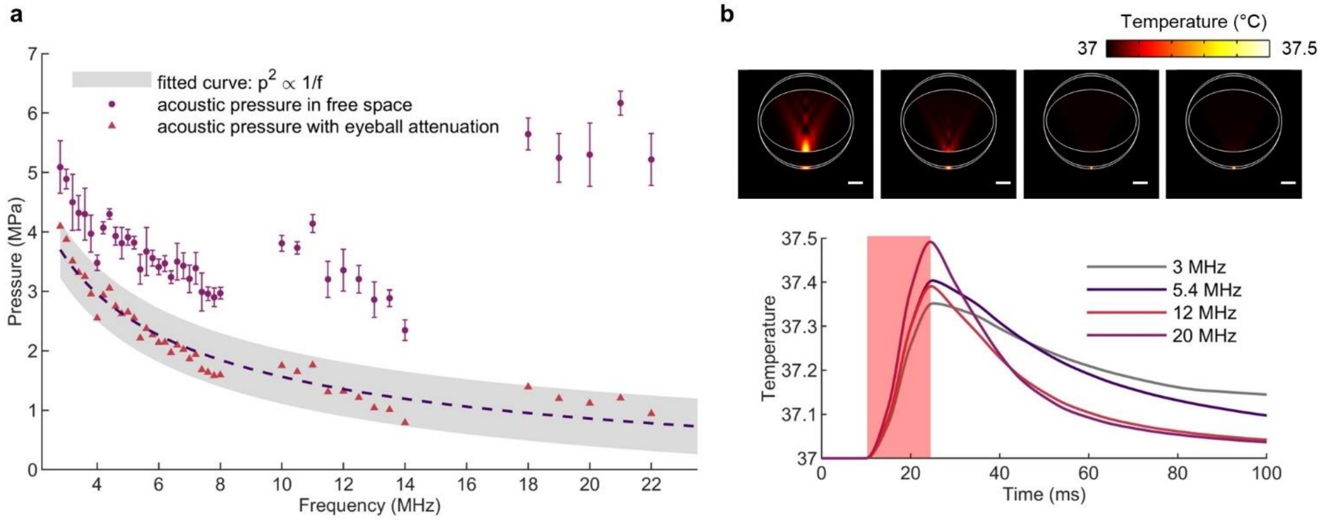

Supplementary Fig. 15. **ARF is the in vivo physical mechanism of ultrasound retina stimulation. a,** Frequency-dependent acoustic pressure threshold that could consistently evoke neuron responses, and a fitted curve indicating the inverse proportional relationship between the square of pressure threshold and frequency. n = 8 animals; Mean, s.d. **b,** Ultrasound-induced temperature increase in ex-vivo eyeball at different center frequencies. The ultrasound pulse length was 15 ms. White bars indicate the length of 1 mm.

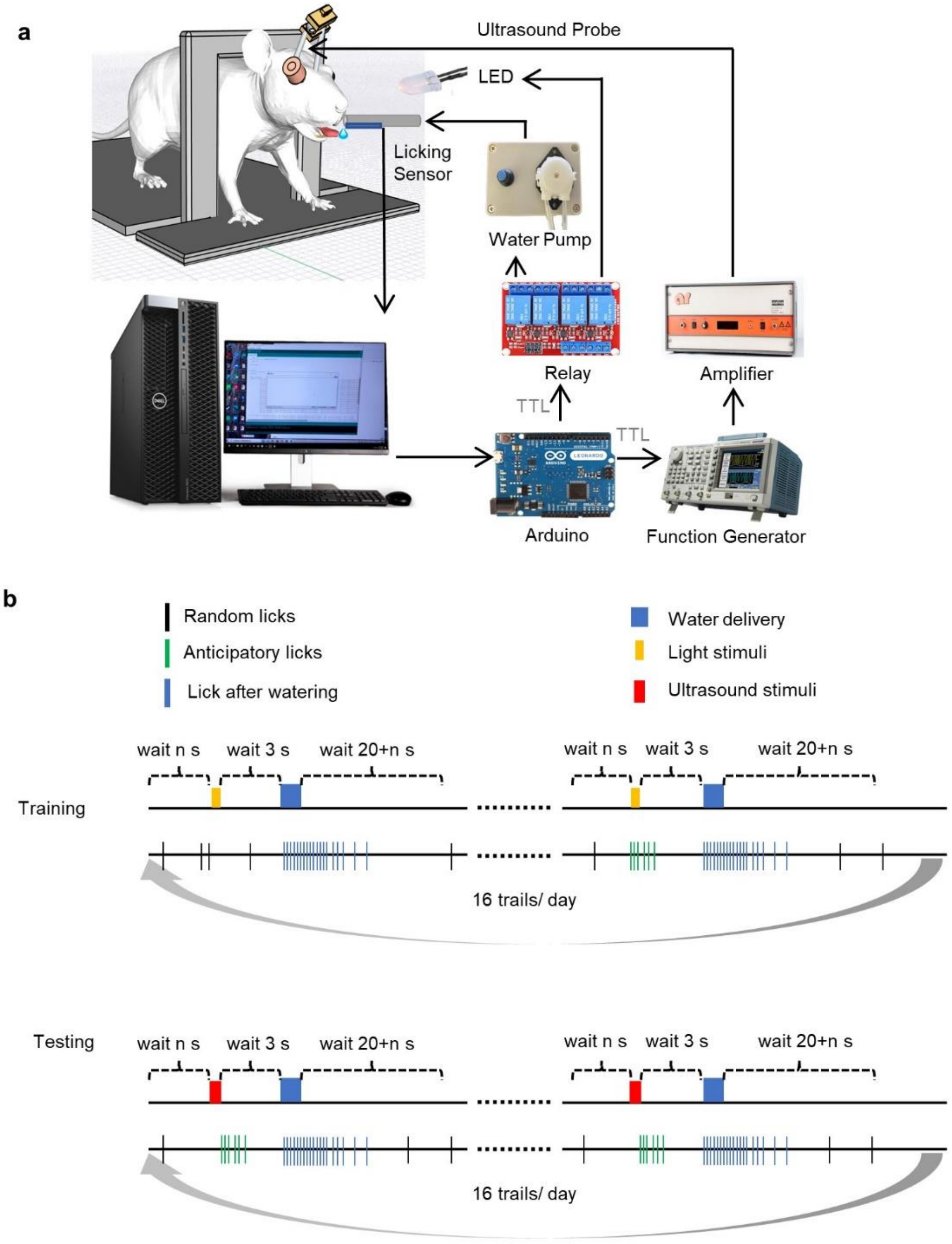

Supplementary Fig. 16. Details of water-licking behavior experiment. **a,** The experiment setup of the water-licking behavior test. **b,** The time sequences of stimulation and observation in training and testing stages.

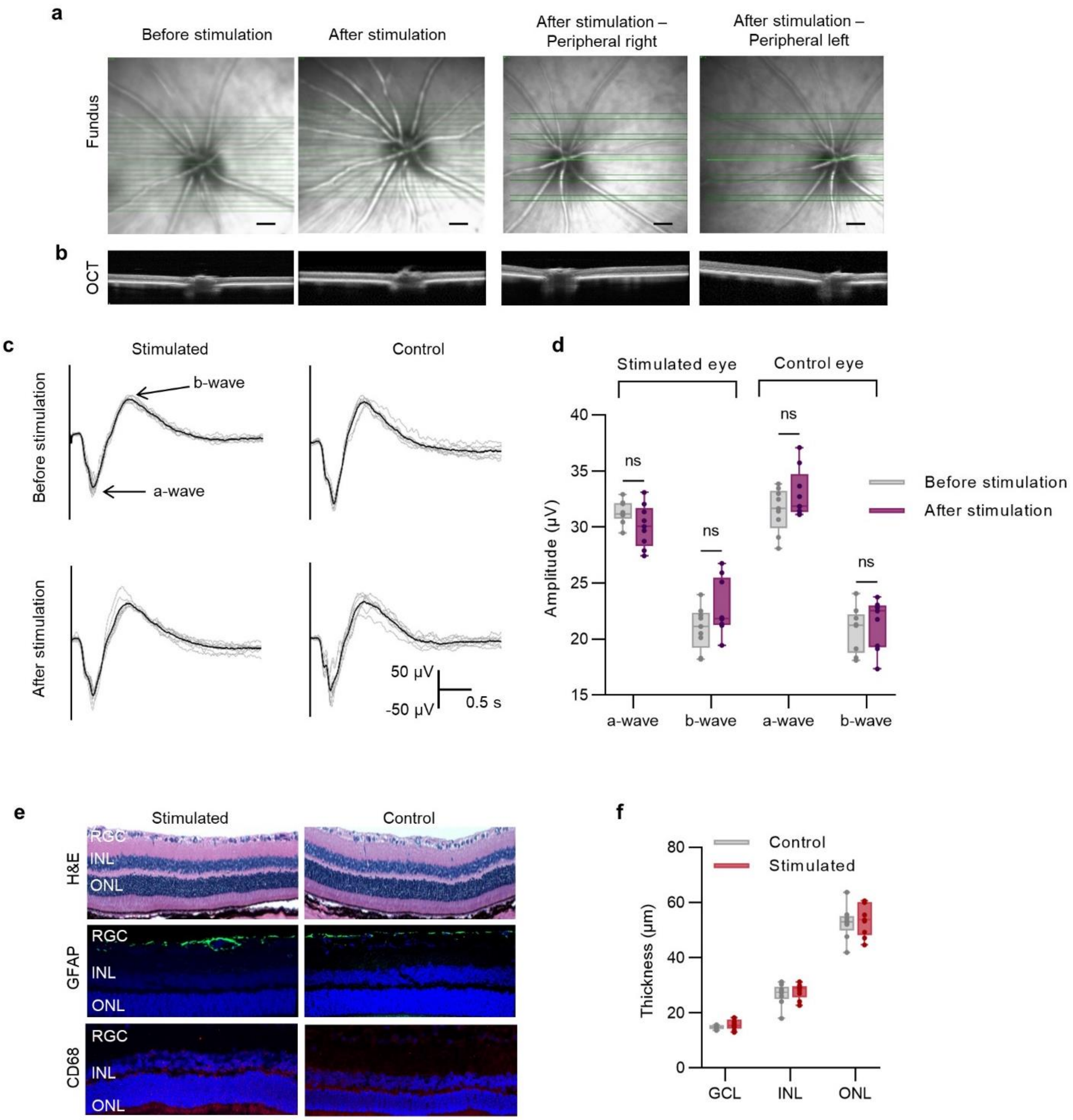

Supplementary Fig. 17. **U-RP is safe in long-term use. a,** In vivo fundus imaging of retina before and after the ultrasound stimulation. Peripheral parts (right and left) of the retina were also imaged. Black bars indicate the length of 1 mm. **b,** In vivo OCT imaging of retina before and after the ultrasound stimulation. Peripheral parts (right and left) of the retina were also imaged. **c,** In vivo ERG measurement of photoreceptors and bipolar cells function before and after the ultrasound stimulation. In the control group, the ultrasound stimulation was focused between the eye and ear of the rat. **d,** Statistical analysis of signal amplitudes of ERG. $n = 9$ from three healthy rats. $p = 0.596$, 0.201, 0.425, 0.965 from two-way ANOVA with Sidak multiple comparison. **e,** Histology analysis of the ex vivo retina after the ultrasound stimulation. Similar results were repeated independently on three healthy rats. Top: H&E staining. Mid: GFAP staining. Bot: CD68 and IBA1 staining. **f.** Statistical comparison of ultrasound retina layer thickness of ganglion cell layer (GCL), inner nuclear layer (INL), outer nuclear layer (ONL). $n = 9$; The box extends from the 25th to 75th percentiles and the line in the middle of the box is plotted at the median. Whiskers cover from the minimum to maximum and all data points. Source data are provided as a Source Data file.

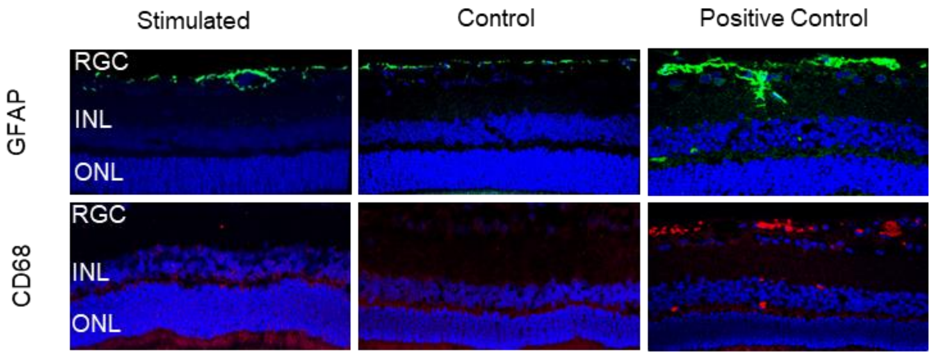

Supplementary Fig. 18. Immunostaining analysis of the ex vivo retina after the ultrasound stimulation with positive control group. An inflammatory response in the retina was intentionally induced in the positive control group.

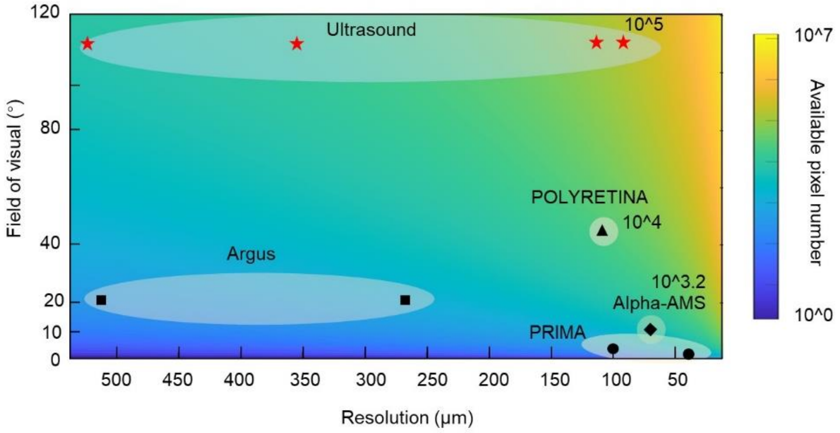

Supplementary Fig. 19. **A summary of resolution and field of view of existing electrode-based retina prostheses and U-RP.**

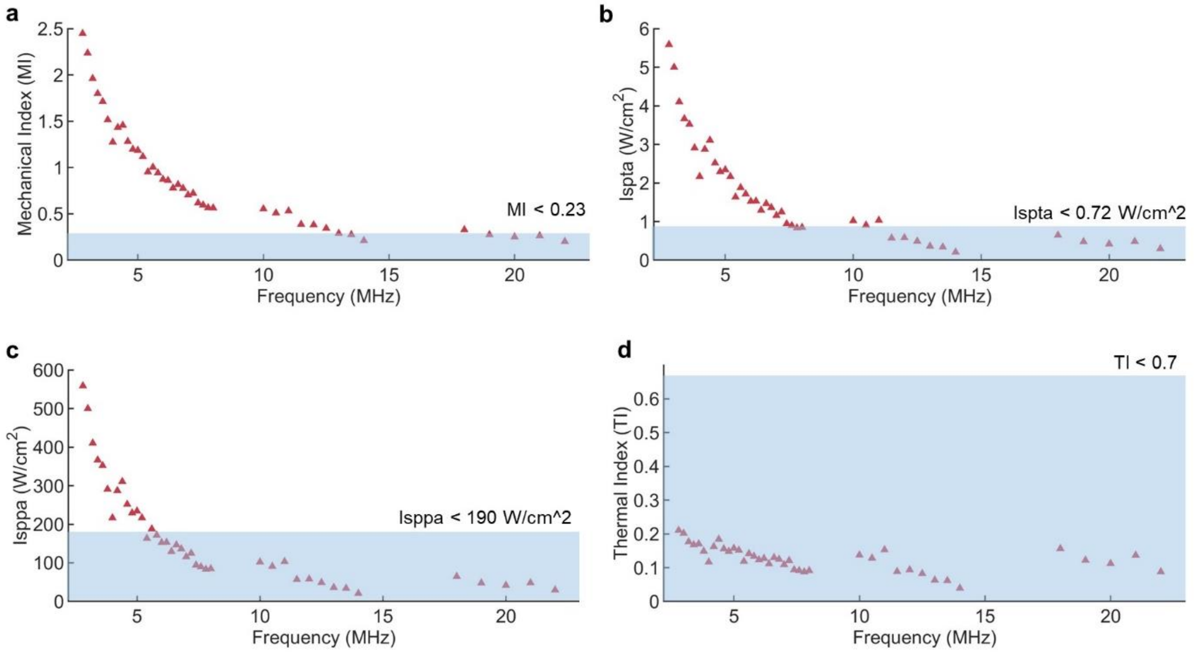

Supplementary Fig. 20. **Ultrasound safety indexes of U-RP. a,** The frequency-dependent mechanical index. **b,** The frequency-dependent spatial peak temporal average intensity (I_{spta}). **c,** The frequency-dependent spatial peak pulse average intensity (I_{sppa}). **d,** The frequency-dependent thermal index. Blue regions indicate the parameters that are within the FDA requirements for eye imaging.

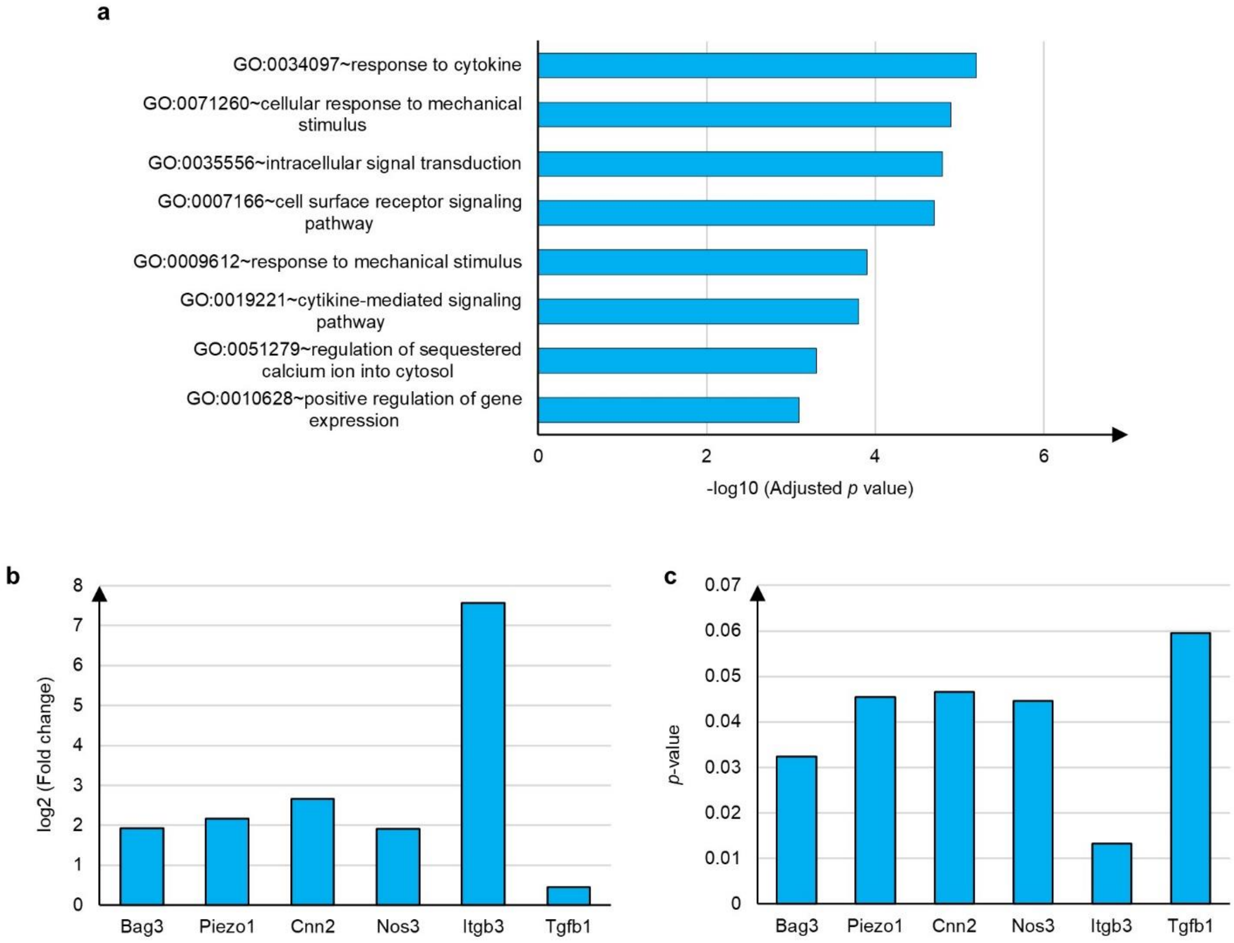

Supplementary Fig. 21. Comparison of gene expression between pre and after US stimulation group was performed. Using DESeq2, the Wald test was performed to generate p-values and log2 fold changes. Genes with a p-value < 0.05 and absolute log2 fold change > 1 were called as differentially expressed genes. **a,** Gene ontology analysis. Differentially expressed genes were clustered by their gene ontology and the enrichment of gene ontology terms was tested using Fisher exact test (GeneSCF v1.1-p2). **b,** The log2 fold change of each gene in group GO:0071260. **c,** The *p*-value of each gene in group GO:0071260.

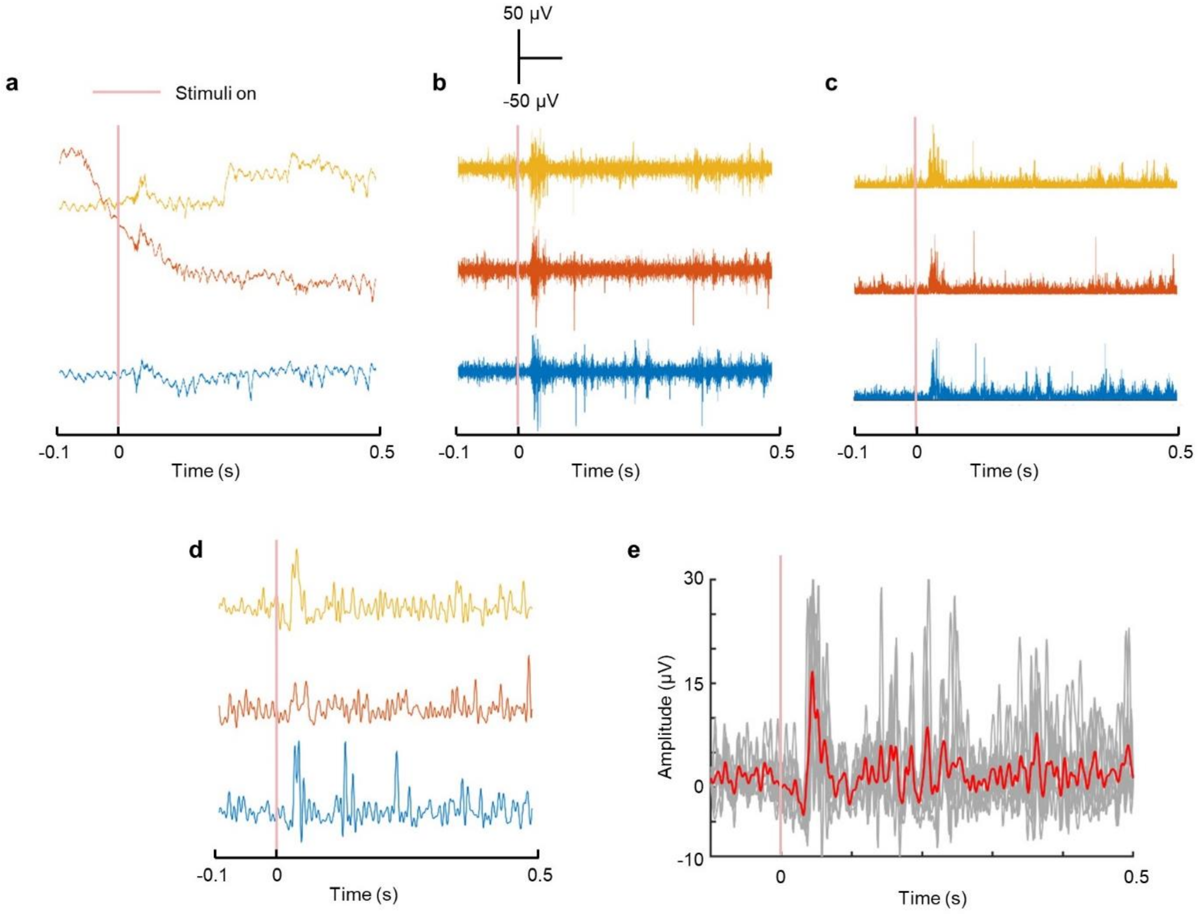

Supplementary Fig. 22. Procedures of signal processing sequence. **a,** Three representative repetitions of raw signals acquired by an electrode of MEA. **b,** Signals after highpass filter. **c,** Signals after rectification. **d,** Signals after rectification. **e,** Signals after down sampling and lowpass filter**.** Pink solid lines indicate the beginning of ultrasound stimulation.
