# Supplementary material for: Noninvasive imaging-guided ultrasonic neurostimulation with arbitrary 2D patterns and its application for high-quality vision restoration
Source: Nat Commun. 2024 May 27;15:4481. doi: 10.1038/s41467-024-48683-6 (PMC11130148; doi:10.1038/s41467-024-48683-6)
Supplement: Supplementary file 3 — Reporting Summary [file 41467_2024_48683_MOESM3_ESM.pdf]

nature portfolio

Corresponding author(s): Qifa Zhou

Last updated by author(s): Apr 17, 2024

Reporting Summary

Nature Portfolio wishes to improve the reproducibility of the work that we publish. This form provides structure for consistency and transparency in reporting. For further information on Nature Portfolio policies, see our Editorial Policies and the Editorial Policy Checklist.

Statistics

For all statistical analyses, confirm that the following items are present in the figure legend, table legend, main text, or Methods section.

n/a	Confirmed	
☐	☒	The exact sample size (*n*) for each experimental group/condition, given as a discrete number and unit of measurement
☐	☒	A statement on whether measurements were taken from distinct samples or whether the same sample was measured repeatedly
☐	☒	The statistical test(s) used AND whether they are one- or two-sided<br>*Only common tests should be described solely by name; describe more complex techniques in the Methods section.*
☒	☐	A description of all covariates tested
☒	☐	A description of any assumptions or corrections, such as tests of normality and adjustment for multiple comparisons
☐	☒	A full description of the statistical parameters including central tendency (e.g. means) or other basic estimates (e.g. regression coefficient) AND variation (e.g. standard deviation) or associated estimates of uncertainty (e.g. confidence intervals)
☐	☒	For null hypothesis testing, the test statistic (e.g. *F*, *t*, *r*) with confidence intervals, effect sizes, degrees of freedom and *P* value noted<br>*Give P values as exact values whenever suitable.*
☒	☐	For Bayesian analysis, information on the choice of priors and Markov chain Monte Carlo settings
☒	☐	For hierarchical and complex designs, identification of the appropriate level for tests and full reporting of outcomes
☒	☐	Estimates of effect sizes (e.g. Cohen's *d*, Pearson's *r*), indicating how they were calculated

Our web collection on statistics for biologists contains articles on many of the points above.

Software and code

Policy information about availability of computer code

Data collection	64-channel Lablynx recording system (Neuralynx, Bozeman, MT, USA)
Data analysis	Matlab 2019b, GraphPad Prism 9, https://github.com/gengxilu/UltrasoundRP.git (10.5281/zenodo.10989656)

For manuscripts utilizing custom algorithms or software that are central to the research but not yet described in published literature, software must be made available to editors and reviewers. We strongly encourage code deposition in a community repository (e.g. GitHub). See the Nature Portfolio guidelines for submitting code & software for further information.

Data

Policy information about availability of data

All manuscripts must include a data availability statement. This statement should provide the following information, where applicable:

- Accession codes, unique identifiers, or web links for publicly available datasets
- A description of any restrictions on data availability
- For clinical datasets or third party data, please ensure that the statement adheres to our policy

A sample data of raw neuron activities in this study have been deposited in the https://figshare.com/articles/dataset/UltrasoundRP/25438252. All data generated in this study are provided in the Supplementary Information/Source Data file. Any additional requests for information can be directed to, and will be fulfilled by, the corresponding authors. Source data are provided with this paper.

Research involving human participants, their data, or biological material

Policy information about studies with human participants or human data. See also policy information about sex, gender (identity/presentation), and sexual orientation and race, ethnicity and racism.

Reporting on sex and gender	N/A
Reporting on race, ethnicity, or other socially relevant groupings	N/A
Population characteristics	N/A
Recruitment	N/A
Ethics oversight	N/A

Note that full information on the approval of the study protocol must also be provided in the manuscript.

Field-specific reporting

Please select the one below that is the best fit for your research. If you are not sure, read the appropriate sections before making your selection.

☒ Life sciences ☐ Behavioural & social sciences ☐ Ecological, evolutionary & environmental sciences

For a reference copy of the document with all sections, see nature.com/documents/nr-reporting-summary-flat.pdf

Life sciences study design

All studies must disclose on these points even when the disclosure is negative.

Sample size	Sample size was determined based on the preliminary results.
Data exclusions	No data was excluded.
Replication	All replications are successful. Replication on different animals were performed every 2 days. Stimulation on each animal was repeated every 6 seconds.
Randomization	This is not relevant to our study because the retina function difference among rats and the vision difference between left and right eyes are not within the interest of the study. Any rat that meets the experiment requirements is used and the choice of stimulating left or right eyes are totally random.
Blinding	This is not relevant to this study because the results are dependent on the real-time neural signals instead of statistical difference between animals.

Reporting for specific materials, systems and methods

We require information from authors about some types of materials, experimental systems and methods used in many studies. Here, indicate whether each material, system or method listed is relevant to your study. If you are not sure if a list item applies to your research, read the appropriate section before selecting a response.

Materials & experimental systems

n/a	Involved in the study	
☐	☒	Antibodies
☒	☐	Eukaryotic cell lines
☒	☐	Palaeontology and archaeology
☐	☒	Animals and other organisms
☒	☐	Clinical data
☒	☐	Dual use research of concern
☒	☐	Plants

Methods

n/a	Involved in the study	
☒	☐	ChIP-seq
☒	☐	Flow cytometry
☒	☐	MRI-based neuroimaging

Antibodies

Antibodies used	GFAP- mAb #3670, CD68, PIMA513324, Mouse anti-Rat, CD68 Monoclonal Antibody (KP1), Invitrogen™, USA
Validation	GFAP: After several phosphate-buffered saline (PBS) washes, slides were incubated for at least 1hr at room temperature in fluorescent-labeled secondary antibody (Alexa Fluor 488 goat anti-rabbit IgG (H + L) (dilution of 1:300 (Thermo Scientific, USA). After staining, the slides were mounted with a fluorescent-enhanced mounting medium with 4′,6-diamidino-2-phenylindole (DAPI) (Vector Laboratory, Burlingame, CA, USA).<br>CD68: After several phosphate-buffered saline (PBS) washes, slides were incubated for at least 1hr at room temperature in fluorescent-labeled secondary antibody (DyLight™ 594, Goat anti-Mouse IgG (H+L) Secondary Antibody, PI35510, Invitrogen™, USA). After staining, the slides were mounted with a fluorescent-enhanced mounting medium with 4′,6-diamidino-2-phenylindole (DAPI) (Vector Laboratory, Burlingame, CA, USA).

Animals and other research organisms

Policy information about studies involving animals; ARRIVE guidelines recommended for reporting animal research, and Sex and Gender in Research

Laboratory animals	Long-Evans rats, Royal College of Surgeon blind rats. male, over 16 weeks of age
Wild animals	Not involved
Reporting on sex	Male
Field-collected samples	Not involved
Ethics oversight	University of Southern California (USC) Institutional Animal Care and Use Committee (IACUC)

Note that full information on the approval of the study protocol must also be provided in the manuscript.

Plants

Seed stocks	*Report on the source of all seed stocks or other plant material used. If applicable, state the seed stock centre and catalogue number. If plant specimens were collected from the field, describe the collection location, date and sampling procedures.*
Novel plant genotypes	*Describe the methods by which all novel plant genotypes were produced. This includes those generated by transgenic approaches, gene editing, chemical/radiation-based mutagenesis and hybridization. For transgenic lines, describe the transformation method, the number of independent lines analyzed and the generation upon which experiments were performed. For gene-edited lines, describe the editor used, the endogenous sequence targeted for editing, the targeting guide RNA sequence (if applicable) and how the editor was applied.*
Authentication	*Describe any authentication procedures for each seed stock used or novel genotype generated. Describe any experiments used to assess the effect of a mutation and, where applicable, how potential secondary effects (e.g. second site T-DNA insertions, mosiacism, off-target gene editing) were examined.*
